# *Inula salicina* L.: Insights into Its Polyphenolic Constituents and Biological Activity

**DOI:** 10.3390/ph17070844

**Published:** 2024-06-27

**Authors:** Viktoria Ivanova, Paraskev Nedialkov, Petya Dimitrova, Tsvetelina Paunova-Krasteva, Antoaneta Trendafilova

**Affiliations:** 1 Institute of Organic Chemistry with Centre of Phytochemistry, Bulgarian Academy of Sciences, 1113 Sofia, Bulgaria; viktoria.genova@orgchm.bas.bg; 2 Pharmacognosy Department, Faculty of Pharmacy, Medical University of Sofia, 1000 Sofia, Bulgaria; pnedialkov@pharmfac.mu-sofia.bg; 3 Stephan Angeloff Institute of Microbiology, Bulgarian Academy of Sciences, 1113 Sofia, Bulgaria; pdimitrova998@gmail.com (P.D.); pauny@abv.bg (T.P.-K.)

**Keywords:** *Inula salicina* L., UHPLC-MS/MS, phenolics, flavonoids, flavoalkaloid, antioxidant potential, sun protection factor, bacterial biofilms

## Abstract

In this study, UHPLC-HRMS analysis of the defatted methanol extract obtained from *Inula salicina* L. led to the identification of 58 compounds—hydroxycinnamic and hydroxybenzoic acids and their glycosides, acylquinic and caffeoylhexaric acids, and flavonoids and their glycosides. In addition, a new natural compound, N-(8-methylnepetin)-3-hydroxypiperidin-2-one was isolated and its structure was elucidated by NMR spectroscopy. The presence of a flavoalkaloid in genus *Inula* is described now for the first time. Chlorogenic acid was the main compound followed by 3,5-, 1,5- and 4,5-dicaffeoylquinic acids. The methanol extract was studied for its antioxidant potential by DPPH, ABTS, and FRAP assays and sun protective properties. In addition, a study was conducted to assess the effectiveness of the tested extract in inhibiting biofilm formation by Gram-positive and Gram-negative strains. Results from crystal violet tests revealed a notable decrease in biofilm mass due to the extract. The anti-biofilm efficacy was confirmed through the observation of the biofilm viability by live/dead staining. The obtained results showed that this plant extract could be used in the development of cosmetic products with antibacterial and sun protection properties.

## 1. Introduction

Plants are a great source of compounds with distinct pharmacological properties that are effectively used in both traditional and official medicine for treatment of various diseases [1]. Oxygen metabolism is principal for human life, but it is also responsible for the production of reactive oxygen and reactive nitrogen species, which significantly affect the regulation of biological processes and cell functions [2]. Reactive oxygen species in the body often lead to an increase in the oxidative stress and the development of many chronic diseases such as cardiovascular, cancer, diabetes, obesity, etc.

Long exposure to UV-B (320–280 nm) radiation is mainly responsible for inducing the skin problems and increases the risk of skin diseases (cancer and photoallergic reactions) [3]. Using skin protectors is one way to stop or reduce UV radiation. In recent years, various plant extracts have been described as an alternative to synthetic sunscreens due to the content of phenolic compounds that possess absorption in the UV region and good antioxidant properties [4,5,6,7,8].

Biofilm is a highly effective microbial community resistant to antimicrobials or disinfectants posing significant challenges related to biofilm contamination on biotic or abiotic surfaces. Moreover, the contamination, difficult removal, and high tolerance of biofilms to antibiotics significantly increase the occurrence of infections, patient morbidity, hospitalization, or mortality rates [9]. Future efforts to control biofilm infections should focus on investigating the therapeutic potential of various inhibitors, including extracts from medicinal plants, and testing their anti-biofilm effects. The abundance of Bulgarian medicinal plants represents a rich yet largely unexplored resource for potential biofilm inhibitors. While many plant extracts are acknowledged for their effectiveness in modulating bacterial quorum sensing [10], the extent of their application as potential biofilm inhibitors remains an area of ongoing exploration.

The genus *Inula* (Asteraceae) is represented by approximately 100 species spread out mainly in Europe and Asia. Species from this genus are used in the traditional medicine (including traditional Chinese medicine) for the treatment of asthma, bronchitis, digestive disorders, urinary tract infections, and also skin [11,12]. Species of genus *Inula* possess various pharmacological properties, including anti-inflammatory, anti-allergic, anti-oxidative, anti-tumor, antimicrobial, anti-diabetic, gastroprotective, hepatoprotective, neuroprotective, cardioprotective, anti-aging, etc. [11,12,13,14,15], due to the biologically active compounds they contain, such as sesquiterpene lactones, flavonoids, mono- and dicaffeoyl esters of quinic acid, etc. [11,12,15,16,17].

*Inula salicina* L. (Irish fleabane, willow-leaved yellowhead) is distributed across Eurasia from Portugal to Japan [18,19]. The plant is used in folk medicine to treat angina, hernias, skin rashes, warts, and its leaves are useful as a wound healing agent [20] as well as a herbal tea in Spain [21]. The healing properties of this plant have not been clearly proven yet. The literature survey revealed only a few reports on biological activity of *I. salicina*. Thus, Yıldırım et al. reported low to moderate inhibitory activity against acetylcholinesterase, butyrylcholinesterase and α-amylase enzymes of the methanol extract of *I. salicina* ant its *n*-hexane, chloroform, ethyl acetate and aqueous methanol fractions and a good anti-inflammatory activity of the ethyl acetate fraction [22]. In the same study, all extracts had moderate effect against *Candida* species, while the chloroform fraction exhibited a notable antimicrobial activity against *Staphylococcus aureus* and *S. epidermis* [22]. Recently, chloroform and methanol extracts from five *Inula* species, including *I. salicina* have been studied for their anti-biofilm properties and pigment synthesis in *C. violaceum* [23]. Sevindik et al. reported the anti-urease activity of the ethanol extract of *I. salicina* which was better than thiourea (IC_50_ 0.0122 vs. 0.0167 µg/mL) [24]. Different extracts of *I. salicina* from Turkey were also shown to have good to high antioxidant and free radical scavenging activities attributed to the presence of a significant amount of total phenolic compounds (TPC) [22,24]. The literature data regarding the chemical constituents are scarce. So far, chlorogenic and caffeic acids [25], apigenin [25], nepetin [26], hyperoside [25], three thymol derivatives [27], and two sesquiterpene lactones (alantolactone and isoalantolactone) [27] have been reported. In our recent study, no sesquiterpene lactones were detected in the chloroform extract *I. salicina* even in traces [28]. Instead, fifteen triterpene alcohols and their 3-*O*-esters (acetates and palmitates) were identified [28].

Within this context, our objectives were to perform a comprehensive phytochemical characterization of the methanol extract of *I. salicina* aerial parts and to evaluate its in vitro antioxidant, sun protective, and anti-biofilm properties. Through this study, our goal is to gain more in-depth information about the chemical composition of this extract and shed light on its potential health benefits.

## 2. Results and Discussion

### 2.1. Identification of Compounds in Inula salicina by UHPLC-MS/MS and NMR

**Figure 1:**
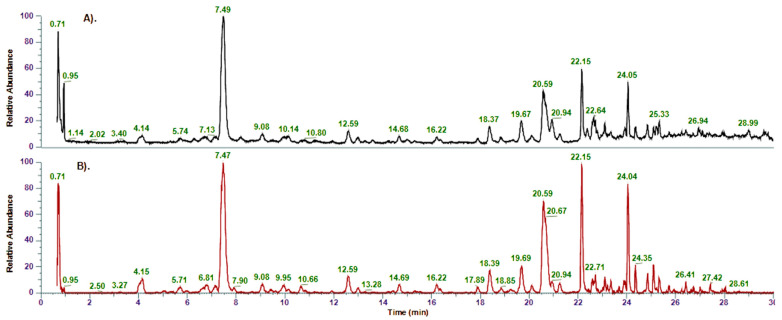
Full-scan LC-MS chromatograms of *Inula salicina*: (**A**). TIC in positive mode; (**B**). Base peak in negative mode.

The methanol extract obtained from *Inula salicina*’s aerial parts was investigated by UHPLC-MS/MS (Figure 1). Since ESI in a negative ionization mode is more sensitive for phenolic compounds, the results from this analysis are presented in Table 1. In total, 58 compounds were identified based on their retention time, *m*/z values, molecular formula, fragmentation pattern and comparison with the literature data [29,30,31,32,33,34,35,36,37,38,39], and open access LC-MS libraries. Of them, 19 compounds were determined by direct comparison with standards and/or by ^1^H NMR (Table 1, Tables S1 and S2, Figure S1) after their isolation and 39 compounds were tentatively identified. The described above compounds belong to following main metabolite classes: hydroxycinnamic and hydroxybenzoic acids and their glycosides (**1**, **2**, **4**, **6, 7**, **9**, **14**, and **21**); acylquinic acids (**3**, **5**, **8**, **10–13**, **15**, **16**, **23**, **25**, **26**, **30**, **31**, **33**, **36–43**, **45**, **49**, **50**); caffeoylhexaric acids (**27**, **32**, **34**, **35**, **44**, **46**, **53**, **54**); and flavonoids and their glycosides (**17–20**, **22**, **24**, **28**, **29**, **47**, **48**, **51**, **52**, **55–58**).

Compound **1** had [M-H]^−^ at *m*/*z* 191 and characteristic for quinic acid fragment ions in its MS/MS [31]. Compounds **2**, **9** and **14** displayed [M-H]^−^ at *m*/*z* 153, 179 and 163 as well as [M-H-CO_2_]^−^ at *m*/*z* 109, 135 and 119 in MS/MS, and were determined as protocatechuic, caffeic and p-coumaric acids, respectively. Compound **7** showed [M-H]^−^ at *m*/*z* 193 and characteristic MS/MS ions at *m*/*z* 178 and 134 as a result of consequent elimination of CH_3_^•^ and CO_2_ (15 and 44 Da, respectively) and was identified as ferulic acid [39]. Compounds **4** and **6** showed [M-H]^−^ at *m*/*z* 341 and MS/MS ion at *m*/*z* 179 indicative for caffeoyl moiety [caffeic acid-H]^−^ as a result from the elimination of 162 Da (hexose) and were tentatively identified as *O*-caffeoyl hexose isomers [31]. Compound **21** had [M-H]^−^ at *m*/*z* 461 and MS/MS ions at *m*/*z* 323 [M-salicylic acid-H]^−^, 179 [caffeic acid-H]^−^, 161 [323-C_6_H_10_O_5-_H]^−^ and 137 [salicylic acid-H]^−^ and was tentatively identified as caffeoyl-(salicyl)-hexoside [38].

Further, 9 mono-, 16 di-, and 1 triacylquinic acids were also recognized by the characteristic for each subclass fragment ions [30,32,33,34,35,36,37,38]. Caffeoyl (**3**, **8**, **10** and **11**), p-coumaroyl (**5**, **12**, **13** and **15**) and feruloyl (**16**) quinic acids were recognized by their deprotonated molecular ion at *m*/*z* 353, 337 and 367, respectively. Further, the characteristic for C-5 substituted quinic acids’ base peak at *m*/*z* 191 in their MS/MS and led to determination of **8**, **13** and **16** as 5-*O*-caffeoyl, 5-O-*p*-coiumaroyl and 5-*O*-feruloylquinic acids, respectively. 3,4-, 3,5-, 1,5- and 4,5-Dicaffeoylquinic acids (**23**, **25**, **26** and **30**) were identified by comparison with authentic standards. The structure of **8**, **25** and **26** was additionally confirmed by ^1^H NMR (Table S1). Compounds **43** and **49** were identified as 3,5- and 4,5-di-*O-p*-coumaroylquinic acids based on their [M-H]^−^ at *m*/*z* 483, a base peak at *m*/*z* 337, a cinnamate-derived peak at *m*/*z* 163 (in **43**) and a diagnostic “dehydrated” quinic acid ion at *m*/*z* 173 (in **49**). Six compounds (**31**, **33**, **36**, **37**, **40** and **41**) displayed the same [M-H]^−^ at *m*/*z* 499 and fragment ions at *m*/*z* 353 and/or 337 with different intensity in their MS/MS spectra, corresponding to the elimination of caffeoyl and *p*-coumaroyl units. Some additional peaks at *m*/*z* 335 [353-H_2_O]^−^ and 319 [337-H_2_O]^−^, and “dehydrated” quinic acid ion at *m*/*z* 173 allowed their identification as *p*-coumaroyl-caffeoylquinic acid isomers [36]. In addition, four feruloyl-caffeoylquinic acids (**38**, **39**, **42** and **45**) were identified by their [M-H]^−^ at *m*/*z* 529 and characteristic fragmentation ions at *m*/*z* 367 [M-C_9_H_6_O_3_-H]^−^, 353 [M-C_10_H_8_O_3_-H]^−^, 349 [367-H_2_O]^−^, and 179 [caffeic acid-H]^−^. 3,4,5-Tricaffeoylquinic acid (**50**) was deduced from its [M-H]^−^ at *m*/z 677 and the fragment ions at *m*/*z* 515, 353 and 191 due to the loss of three caffeoyl units. Their position at C-3, C-4 and C-5 was deduced from the peaks at *m*/*z* 173, 135 and 179 [30].

Four tricaffeoylhexaric acids (**27**, **32**, **34** and **35**) and a tetracaffeoylhexaric acid (**44**) were also detected. Their identification was based on their [M-H]^−^ at *m*/*z* 695 and 857, respectively, the base peak at *m*/*z* 209 [hexaric acid–H]^−^ and diagnostic fragment ions resulting from the sequential elimination of three of four caffeoyl moieties [38]. Compound **46** showed [M-H]^−^ at *m*/*z* 617 and fragment ions at *m*/*z* 293 [M-2caffeoyl-H]^−^ and 191 [M-2caffeoyl-C_5_H_10_O_2_]^−^ due to the loss of two caffeoyl residues and a subsequent loss of 102 Da (2-methylbutiric acid/isovaleric acid). Therefore, compound **46** was tentatively identified as 2-methylbutanoyl/isovaleryl dicaffeoylhexaric acids. In the same manner, compound **54** was determined as 2-methylbutanoyl/isovaleryl tricaffeoylhexaric acid. Similarly, compound **53** was identified as isobutanoyl-tricaffeoylhexaric acid as it had [M-H]^−^ at *m*/*z* 765, fragmentation ions due to the loss of three caffeoyl units and a base peak at *m*/*z* 279 as a result of elimination of 88 Da (C_4_H_8_O_2_) [38].

With exception of chlorogenic (**8**) and caffeic acids (**9**) [25] all other compounds are described now for the first time in *I. salicina*. As far as we know, there are only a few HPLC-MS/MS analyses of *Inula* species so far [29,38,40,41,42,43,44,45]. Among the identified acids and their derivatives, acylquinic acids dominated, especially mono- and dicaffeoyl quinic acids. Caffeoylquinic acid derivatives are common constituents of species of Asteraceae family [46], they could not be used as chemotaxonmical markers, but definitely contributed to the various biological activity of the plant extracts [47,48]. Caffeoylhexaric acids have been reported in *I. sarana* [38] and *I. viscosa* [44] only. However, it seems that these compounds are characteristic for the species of the tribe *Inulae* as they have been detected in some related genera such as *Pulicaria*, *Carpesium*, *Xerolekia*, etc. [49,50,51].

Free aglycones, flavonoid mono- and di-glycosides, including seven flavone and eight flavonol derivatives were recognized by their mass-spectral fragmentation pattern [29,38]. Compounds **47, 48** and **58** were identified as the free aglycones quercetin, luteolin and apigenin by their [M-H]^−^ at *m*/*z* 301, 285 and 269, respectively, as well as by comparison with authentic standards. In addition, five methoxylated aglycones patuletin (**51**), nepetin (**52**), chrysoeriol (**55**), jaceosidin (**56**) and quercetagetin trimethyl ether (**57**) were identified from the corresponding deprotonated molecular ions and the characteristic fragmentations [29,38]. The structures of flavonoids **47, 48, 51, 52** and **58** were also confirmed by ^1^H NMR (Table S2). MS/MS spectra of quercetin glycosides rutin (**17**) and isoquercitrin (**18**) contained a peak at *m*/z 301, characteristic for quercetin and derived by the elimination of 308 (rutinose) and 162 (hexose) Da from the corresponding [M-H]^−^ at *m*/*z* 609 and 463. The structure of **17** and **18** was additionally confirmed by comparison with authentic standards. Compounds **19**, **20**, **22**, **24**, and **29** showed similar fragmentation patterns yielding prominent peaks at *m*/*z* 285 (**19** and **29**), 331 (**20**) and 315 (**22** and **24**) resulting from the elimination of a hexose unit (162 Da) from the precursor ions. The presence of glucopyranosyl moiety and the aglycone part in the structures of **20**, **21**, **23**, and **30** was further confirmed by ^1^H NMR (Table S3) after their isolation from the methanol extract. Compound **24** was tentatively determined as isorhamnetin hexoside. It is worth noting that with the exception of nepetin (**52**) and apigenin (**58**) [25,26], all other identified flavonoids are reported in *I. salicina* for the first time. The detection of 6-methoxyflavones and 6-methoxyflavonols in *I. salicina* is not surprising, as these compounds have been previously found in many *Inula* species [11,16,52,53,54,55] and they can be considered as a chemotaxonomic characteristic at the genus level.

**Figure 2:**
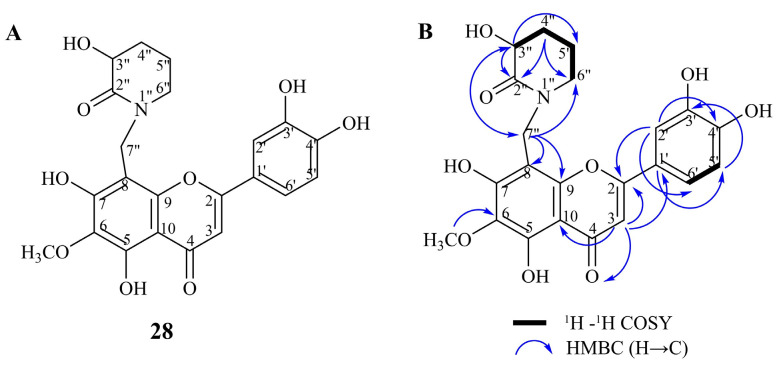
Structure (**A**) and key HMBC and ^1^H-^1^H COSY correlations (**B**) of N-(8-methylnepetin)-3-hydroxypiperidin-2-one (**28**).

Finally, the low-intensive deprotonated molecular ion [M-H]^−^ at *m*/*z* 442.1150 and the base peak at *m*/*z* 327 [M-C_5_H_8_O_2_N-H]^−^ suggested a flavonoid-alkaloid structure of compound **28** (Figure 2A). The lack of information in the literature and open access LC-MS libraries prompted us to perform further isolation of this compound and elucidate its structure. The HRESIMS in the positive mode of **28** showed [M + H]^+^ at *m*/*z* 444.1284 (calculated for C_22_H_22_O_9_N) pointing out a molecular formula C_22_H_21_O_9_N.

The ^1^H NMR spectrum (Table 2) contained two doublets at δ_H_ 6.94 (*J =* 2.2 Hz) and 7.62 (*J =* 8.4 Hz), a signal at δ_H_ 7.45 dd (*J =* 2.2 and 8.4 Hz), a singlet at δ_H_ 6.59, a methoxyl group signal at δ 3.89. These spectral data were very similar to that of nepetin (6-methoxyluteolin, **52**) suggesting the same aglycone. Surprisingly, instead of the characteristic H-8 signal, additional signals for a benzyl methylene group (δ_H_ 4.72 and 4.58, δ_C_ 47.4) appeared in the ^1^H and ^13^C NMR. The observed HMBC correlations (Figure 2B) of these signals with C-8 (δ_C_ 97.3) and C-9 (δ_C_ 152.5) suggested a C-bound aliphatic portion attached to C-8. Further, ^1^H NMR, HSQC and HMBC spectra contained additional signals for three methylene groups (δ_H_ 3.57/3.36 and δ_C_ 53.4, δ_H_ 2.09/1.95 and δ_C_ 23.0, δ_H_ 2.48/2.18 and δ_C_ 28.8), a hydroxymethine group (δ_H_ 4.07 and δ_C_ 69.3) and a carbonyl group (δ_C_ 172.4). The observed COSY interactions H-3″/H-4″, H4″/H5″ and H5″/H6″ and HMBC correlations H-3″/C-2″, H-3″/C-5″, H-4″/C-2″, H-4″/C-6″ (Figure 2B) confirmed the proposed connectivity of this part of the molecule.

Further, the presence of 3-hydroxypiperidin-2-one moiety was confirmed by the base peak at *m*/*z* 329 in the MS/MS spectrum due to the loss of C_5_H_8_O_2_N unit (114 Da). The observed long range HMBC correlations (Figure 2B) of H-7″ with C-3″and C-6″ showed that the nitrogen atom is attached to C-2″, C-6″ and C-7″. Therefore, the new compound **28** was identified as N-(8-methylnepetin)-3-hydroxypiperidin-2-one. To the best of our knowledge, there is only one report for natural compounds with N-methyl-3-hydroxypiperidin-2-one residue in their structure. These compounds were two flavonol glycosides isolated from *Astragalus monspessulantus* from Bulgaria [56]. This is the first report of the presence of flavoalkaloids in the genus *Inula*. Flavoalkaloids are a unique group of structurally diverse secondary metabolites, consisting of a nitrogen-containing moiety attached to a flavonoid backbone at C-6 or C-8 positions [57], and are considered to be compounds with potential against various diseases such as cancer, inflammation, viral infections, etc. [58].

### 2.2. Quantitative Determination of Total Phenolics, Total Flavonoids, Chlorogenic and Dicaffeoylquinic Acids

Spectrophotometric methods are widely used to assess the total phenolic (TPC) and total flavonoid (TFC) content of the plant extracts as these compounds contribute significantly to their biological activity. In this study, TPC and TFC were found to be 215.57 ± 2.52 mg GAE/g DE and 87.44 ± 0.52 mg CE/g DE (Table 3). The results obtained for TPC were significantly higher than those found for the methanol extract of *I. salicina* from Turkey (143.80 mg GAE/g DE) and its n-hexane, chloroform, and aqueous methanol fractions (40.62, 166.20 and 126.10 mg GAE/g DE) and were twice lower than the ethyl acetate fraction (574.80 mg GAE/g DE) [22]. The TFC of the same extracts varied between 10.22 and 201.40 mg QE/g DE and increased in the following order: n-hexane > chloroform > methanol > aqueous methanol > ethyl acetate [22]. There is only one more study on TPC (58.54 μg GAE/mL) and no other data on TFC of *I. salicina* [24]. In our previous study on six *Inula* species, the TPC of the methanol extracts varied from 28.81 to 119.92 mg GAE/g DE with the highest phenolic level observed in the flower extract of *I. ensifolia* [17]. The study of methanol extracts of *I. britannica* from 11 different Bulgarian habitats revealed high variations in TPC (85.35–141.01mg GAE/g DE) and TFC (19.66–36.80 mg CE/g DE) [45]. Therefore, it can be concluded that the extract of *I. salicina* is the richest in phenolics and flavonoids among the representatives of the genus *Inula* growing in Bulgaria studied so far.

**Figure 3:**
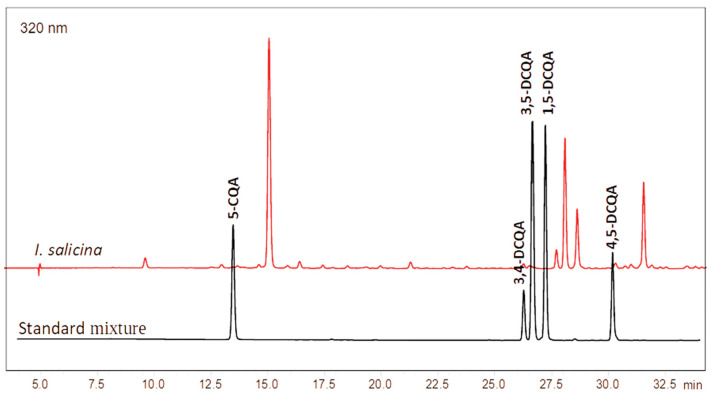
HPLC chromatogram of *I. salicina* extract and standard mixture at 320 nm, 5-CQA–chlorogenic acid, DCQA–dicaffeoylquinic acid.

Further, the content of chlorogenic acid and four dicaffeoylquinic acid (DCQA) isomers was determined by the HPLC method (Figure 3). Chlorogenic acid (5-CQA) was the major component detected in the highest amount (103.39 ± 1.30 mg/g DE), while that of dicaffeoyl esters was significantly lower decreasing in the order 3,5-DCQA > 4,5-DCQA > 1,5-DCQA > 3,4-DCQA (Table 3).

In the study of some Hungarian *Inula* species, it has been reported that *I. salicina* was the richest in chlorogenic acid [25]. In addition, the ray florets of *I. salicina* were found to contain more chlorogenic acid than the disk florets unlike the other studied *Inula* species. It was interesting to compare the results of this study with those found for other *Inula* species growing in Bulgaria [17,45]. Thus, the amount of chlorogenic acid in *I. aschersoniana* var. *aschersoniana*, *I. bifrons*, *I. conyza*, *I. ensifolia*, *I. germanica*, and *I. oculus-christi* was significantly lower (5.48–28.44 mg/g DE) and *I. ensifolia* was the species with the highest amount detected [17]. The content of chlorogenic acid in the extracts of different *I. britannica* populations was also variable (14.99–51.41 mg/g DE) [45]. In these studies, the content of 5-CQA in the respective plants was lower than the total amount of dicaffeoyl esters of quinic acid [17]. Despite the different quantities of the individual DCQAs, 1,5-DCQA was the principal component in all studied species with exception of *I. conyza* [17]. 3,5-DCQA was the major compound in *I. conyza* [17] as the currently studied *I. salicina*. The latter contained a significant amount of 4,5-DCQA, similarly to *I. ensifolia* and *I. oculus-christi.* Therefore, it can be concluded that the extract of *I. salicina* is the richest in chlorogenic acid among the representatives of the genus *Inula* growing in Bulgaria studied so far.

### 2.3. Antioxidant Potential of Inula salicina Extract

DPPH radical scavenging, ABTS radical-ion and FRAP assays, based on different mode of actions are widely used for a preliminary study of the antioxidant potential of various compounds and plant extracts [59]. Thus, the antioxidant capacity of the studied extract measured by the DPPH and ABTS methods was 0.741 ± 0.006 and 0.711 ± 0.007 mM TE/g DE, respectively, while the ferric ion reducing antioxidant power in FRAP assay was found to be 5.77 ± 0.08 µM Fe^2+^/g DE). In a recent study on the antioxidant activity of *Inula salicina* from Turkey it has been found that the ethyl acetate fraction possessed the best DPPH and ABTS activity among the other fractions and the methanol extract and the measured IC_50_ in both assays was similar to that of known standards Trolox and ascorbic acid and better than the synthetic antioxidant BHA [22]. This is the first report on FRAP activity of *I. salicina*. There are reports in the scientific literature regarding antioxidant activity (DPPH, ABTS, FRAP, etc.) of other representatives of genus *Inula* [13,14,17,45,54,60,61]. Unfortunately, slight differences in the assays, measuring units and other factors make it difficult to compare the obtained data. However, it can be concluded that the *Inula* extracts which are richer in polyphenolics are better antioxidants.

### 2.4. In Vitro Sun Protection Factor (SPF) of Inula salicina Extract

**Figure 4:**
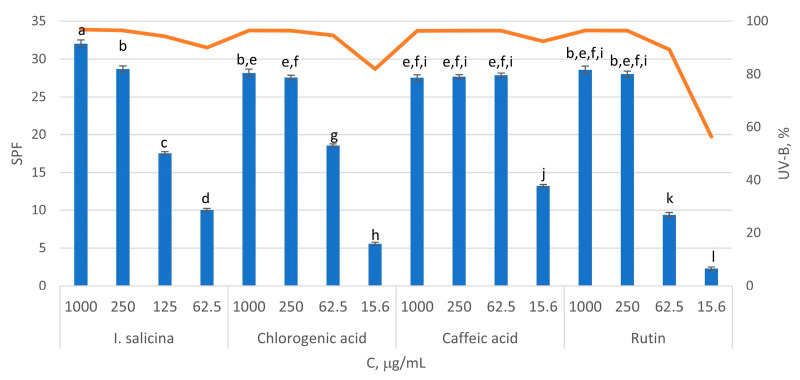
Sun protection factor (SPF) and UV-B absorption (line in orange) of *I. salicina* extract, chlorogenic and caffeic acids and rutin. Subscripts of different letters symbolize a statistically significant difference between the samples (*p* < 0.05).

The methanol extracts of *I. salicina*, chlorogenic and caffeic acids and rutin in four different concentrations were studied for their sun protection factor (SPF) (Figure 4) using the method described by Mansur [62]. As can be seen, all tested samples possessed good SPF at concentrations > 250 µg/mL (SPF 28–32), corresponding to 94–98% UV-B protection [4]. Further, *I. salicina* extract in concentration of 62.5 µg/mL had a SPF 10, corresponding to 90% UV-B protection; it was similar to that of rutin (SPF 9, 89% UV-B) and lower than that of chlorogenic and caffeic acids (SPF 18 and 28, 95 and 96%, respectively).

It can be assumed that phenolic compounds, particularly chlorogenic acid are responsible for the sun protective properties of *I. salicina* methanol extract [3,5,8]. It is worth mentioning that this is the first report on SPF of *Inula* species.

### 2.5. Biofilm Inhibition of Inula salicina Extract

To estimate the ability of *I. salicina* methanol extract to inhibit biofilm formation a crystal violet assay was conducted. The Gram-positive and Gram-negative strains utilized in this study are classified by the World Health Organization as a critical priority due to their antimicrobial resistance. It is well known that these strains cause infections such as otitis, sinusitis, infectious wounds, cystic fibrosis, chronic obstructive pulmonary diseases, osteomyelitis, endocarditis, chronic prostatitis, and others [9]. Their role in these diseases underscores the significance of understanding their pathogenesis and developing targeted treatment strategies. The analysis after 24 h interval of biofilm incubation demonstrated the inhibition effects of *I. salicina* extract on bacterial biofilms. The inhibition capacity of the plant extract was expressed as percentage of biofilm inhibition calculated relative to the control sample, which comprised M63 medium along with the corresponding bacterial strain. The highest anti-biofilm activity of a *I. salicina* methanol extract was detected in the *P. aeruginosa* strain (64.9% ± 0.06), followed by inhibition at *S. aureus* 43.4% ± 0.02. The lowest value was reported in *E. coli* (35.0% ± 0.04). These results are consistent with data from our previous study conducted with another Gram-negative strain, *C. violaceum* [23]. This research also underscores the synergistic effect of various extracts of *Inula* species against virulence factors of *C. violaceum* like biofilm formation, violacein production, and swarming motility. Furthermore, the study stands out as one of the few conducted with an *Inula* species, demonstrating the inhibitory efficacy of the tested extracts on biofilm formation and modeling their 3D structure [23]. The obtained results highlight the plant extracts such as *Inula viscosa*, *Betula pendula*, *Galium odoratum*, *Urtica dioca*, etc. are capable of reducing the biofilm formation of *Escherichia coli*, *Candida albicans*, and *Candida glabrata* [63,64,65]. Moreover, our previous study demonstrated the anti-biofilm activities of CaO/chitosan nanocomposites doped with different extracts from the leaves of *A. indica* and *M. azedarach* [66]. The obtained results showed a significant reduction in biofilms by the nanocomposites labeled with plant extracts in the tested model strains *E. coli* and *S. aureus*.

### 2.6. Live/Dead Biofilm Assays

**Figure 5:**
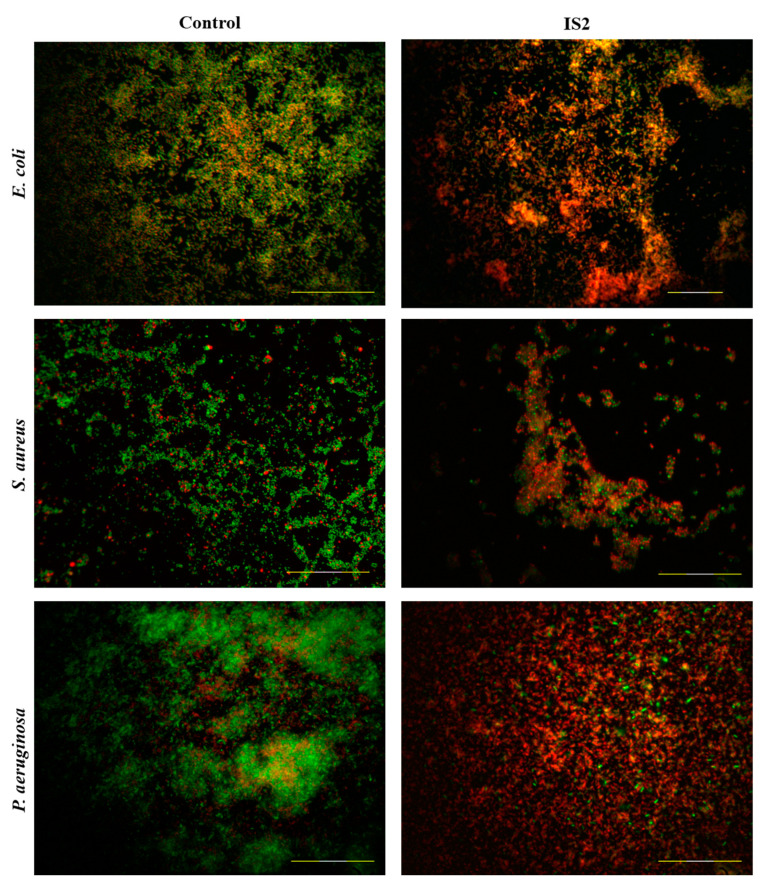
Evaluation of the viability of Gram-positive and Gram-negative bacterial cells within the biofilms. The first column represents the fluorescence microscopy images of *E. coli*, *S. aureus* and *P. aeruginosa* biofilms in the control group. The second column demonstrates the anti-biofilm efficacy of the applied metabolic product of *Inula salicina*-IS2. Bars = 50 μm.

Plant metabolites manifest different mechanisms of action, some of which can lead to anti-bacterial or anti-biofilm effects and subsequently to bacterial cell death [9]. Hence, it is extremely important to see how an extract affects bacterial viability both at the single-cell and biofilm consortium levels. To determine the bacterial cells viability within the biofilms cultivated in the presence of *I. salicina* methanol extract, we applied a live/dead fluorescent viability kit (Figure 5). The *I. salicina* extract is rich in chlorogenic acid and dicaffeoylquinic acids as described above. The scientific data of these metabolic ingredients showed that they have proven antibacterial effect on both Gram-negative and Gram-positive strains [67]. The remarkable anti-biofilm effects of *I. salicina* extract (crystal violet test), especially in *P. aeruginosa,* were also confirmed during the live/dead biofilm staining. In control groups, the formed biofilms consist of live, intact (green) cells. The fluorescence labeling provided in *E. coli* and *S. aureus*, showed that the biofilm is monolayered with the formation of local groups, while in *P. aeruginosa*, the biofilm tends to be multilayered, clustered, and dense, composed mainly of dead cells. Across the three bacterial strains, only single cells remain intact (green) without disruptions in bacterial cell membrane. However, during the assessment of both cell viability and biofilm formation upon the treatment, they are noticeably affected. The biofilm structure is loosened and reduced. The images of *E. coli* and *S. aureus* revealed distinct clustering, aggregation and a reduced biofilm layer, which were consistent with the results from the CV assay. In *E. coli*, the biofilm mainly consisted of non-viable cells, although some green cells were visible in certain areas. Treatment of *S. aureus* biofilms with *I. salicina* extract resulted in substantial cell aggregation, with varying sizes of clusters containing both dead and live cells, mostly in the upper layers.

This may be due to the inability of the phytochemicals to penetrate the dense structure of the biofilm matrix and reach all the bacterial cells. In another study, it was observed that treatment with 2% chlorogenic acid, also present in our extract, resulted in inhibition of the bacterial viability, which indicated cell membrane damage [68]. In contrast, in *P. aeruginosa* biofilm layers disintegrated without forming clusters. A significant part of the observed cells in *P. aeruginosa* were non-viable. However, there was a small portion of intact cells. This may be due to the presence of chlorogenic acid in the extract.

### 2.7. SEM on Pseudomonas aeruginosa Cell Morphology

**Figure 6:**
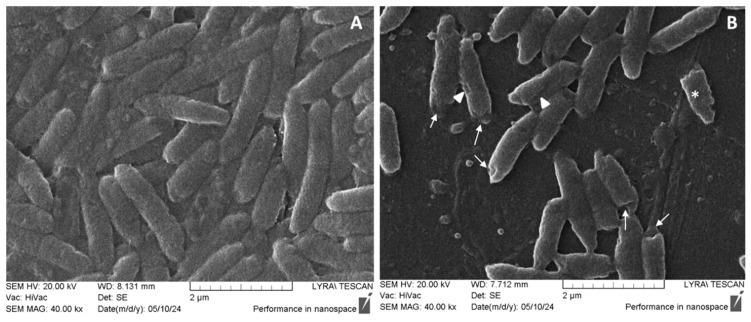
Evaluation of bacterial cell morphology within biofilms by scanning electron microscopy. (**A**) Control group; (**B**) *P. aeruginosa* biofilms incubated with methanol extract from *I. salicina*. Bars = 2 µm. White triangle point to radial indentations, white arrow point to invaginations, white star to disrupted cell wall.

Figure 6 shows the results of the SEM analysis of changes in the biofilm and the morphology of individual bacterial cells after treatment with the methanol extract. The electron microscopic analysis of the biofilms incubated with the extract clearly indicates a significant reduction in biofilm biomass and a lack of multilayer cell distribution compared to the control sample (Figure 6). Moreover, distinct deformations in the surface relief of individual cells are clearly defined (Figure 6B). A notable feature is the destruction of the surface relief with the presence of radial indentations along the length of the cells, indicated by a white triangle. In comparison to the control sample, where no changes in the relief are observed, the treated sample shows widespread invaginations at both poles of the bacterial cells, marked by a white arrow (Figure 6B). An interesting finding is the presence of a cell with a disrupted cell wall and likely release of cytoplasmic content, marked by a white star. Particularly, during the treatment in *P. aeruginosa*, chlorogenic acid can trigger the detachment of lipopolysaccharides from the outer membrane, resulting in high membrane permeability, depolarization, leakage of nutrients and metabolites and ultimately cell death [69]. These scientific data correlate with the SEM results obtained by us when treating the cells with the methanol extract (Figure 6B).

In conclusion, the tested methanol extract can affect both the process of biofilm formation, the cell viability and morphology in tested Gram-positive and Gram-negative strains. The effects varied among the bacterial strains, with an overall reduction in biofilm thickness, multilayer distribution and viability observed. In both Gram-negative strains, *E. coli* and *P. aeruginosa*, the cells were notably morphologically affected and most of them were non-viable. The trend was similar in *S. aureus* except for single intact cells. These differences between the Gram-positive and Gram-negative strains, may be related to the structure of the cell wall, as well as the mechanisms of action of the different bioactive constitutes of the extract and their concentrations [70]. This is once more supported by our viability and SEM assays through which a predominant accumulation of non-viable red and deformed bacterial cells was demonstrated, providing further evidence of the anti-biofilm and also antimicrobial effect of the applied plant extracts. This hypothesis has been confirmed by other researchers where the two active metabolic components—chlorogenic acid and dicaffeoylquinic acid—not only possess antibacterial activity due to their ability to disrupt membrane permeability and potential, but also a strong anti-biofilm effect [71]. Their anti-biofilm activity is probably due to different mechanisms of action. For instance, chlorogenic acid can disturb different processes such as quorum sensing, biofilm formation, bacterial flagella formation, etc. [69,72]. Moreover, it was found that 3,5-dicaffeoylquinic acid exhibits a strong anti-biofilm effect in *P. aeruginosa* [73].

## 3. Materials and Methods

### 3.1. Plant Material

The aerial parts of *Inula salicina* L. were picked up in full flowering stage near Dospat lake, Rhodopes Mts, Bulgaria in 2020, air-dried, grounded and kept in a dark place prior analysis. The species was identified by Assoc. Prof. PhD Ina Aneva (Institute of Biodiversity and Ecosystem Research, Bulgarian Academy of Sciences) according to the following reference specimen (SOM 176701) deposited in the Herbarium of the Institute of Biodiversity and Ecosystem Research, Bulgarian Academy of Sciences.

### 3.2. Preparation of the Methanol Extract

The plant material (54 g) was initially extracted with chloroform (3 × 500 mL) to remove non-polar compounds and chlorophylls followed by an extraction with methanol (3 × 500 mL). All extractions were performed at room temperature for 24 h each. Further filtration and evaporation of the solvent under reduced pressure yielded the methanol extract (4.06 g).

### 3.3. Fractionation of the Methanol Extract and Isolation of Individual Compounds

The fractionation was worked out following the procedure described in [54]. Briefly, a portion of the extract (1.1 g) was re-dissolved in MeOH (10 mL), centrifuged (at 5800 rpm) and a clear methanolic solution was concentrated up to 5 mL. Further CC on Sephadex LH-20 with methanol as an eluent gave two main fractions I (0.62 g) and II (0.39 g). TLC (Silica gel 60 F_254_, EtOAc/HCOOH/CH_3_COOH/H_2_O, 100:11:11:26, spraying with NP reagent (1% diphenylboronic acid 2-aminoethyl ester in ethyl acetate) and UV visualization at 366 nm) of fraction II showed the presence of flavonoids (yellow to orange fluorescence) and caffeoylquinic acids (blue fluorescence) and was further separated by MPLC (LiChroprep RP-18) with H_2_O/CH_3_OH mixtures in different proportions. Next, selected fractions were purified by MPLC (LiChroprep RP-18, CH_3_OH/H_2_O, 50:50) and/or prep. TLC (Silica gel 60 F_254_, CHCl_3_/CH_3_OH, 10:1 or (RP-18, MeOH/H_2_O, 1:1 and 7:3) led to the isolation of 15 compounds: chlorogenic acid (**8**) (6.5 mg), rutin (**17**) (2.3 mg), isoquercitrin (**18**) (1.8 mg), luteolin-7-*O*-glucoside (**19**) (1.2 mg), patulitrin (**20**) (1.1 mg), nepitrin (**22**) (2.2 mg), 3,5-dicaffeoylquinic acid (**25**) (2.1 mg), 1,5-dicaffeoylquinic acid (**26**) (1.8 mg), N-(8-methylnepetin)-3β-hydroxypiperidin-2-one (**28**) (0.8 mg), kaempferol-3-*O*-glucoside (**29**) (1.2 mg), quercetin (**47**) (1.2 mg), luteolin (**48**) (5.6 mg), patuletin (**51**) (0.8 mg), nepetin (6-OMe-luteolin) (**52**) (1.2 mg), and apigenin (**58**) (1.1 mg).

*N-(8-methylnepetin)-3-hydroxypiperidin-2-one (**28**)*:

Yellowish semisolid substance, UV (MeOH): λ_max_ (log ε) 227.5 (2.72), 273.5 (1.80), 349.5 (1.73) nm; HRESIMS: *m*/*z* 444.12841 [M+H]^+^ (calcd. for C_22_H_22_O_9_N, 444.12891, Δ_ppm_ = −1.12); ^1^H and ^13^C NMR: see Table 2.

### 3.4. NMR Analysis

The 1D and 2D NMR (^1^H, COSY, HSQC and HMBC) spectra were recorded on a Bruker Avance II+ 600 NMR spectrometer with operating frequency 600 (^1^H) and 150 (^13^C) using the residual solvent signal (δ_H/C_ 3.31 and 49.3 for CD_3_OD) as a reference. The known compounds were identified by comparison of their ^1^H NMR spectral data (Tables S1–S3) with literature data [34,74,75,76,77,78,79]. ^1^H and ^13^C NMR of compound **28** are presented in Table 2 and Figures S2–S7.

### 3.5. UHPLC-HRMS Analysis

The chromatographic and mass spectrometric conditions of the UHPLC-HRMS analysis were as given in the literature [80] with small modifications. The gradient of the chromatographic separations is given in Table 4. The system was kept at the initial condition for 5 min before each injection. The maximal injection time in full MS mode was set to 80 ms, while stepped normalized collision energy was 10, 15, and 20 (+p mode) as well as 10, 20, and 30 (−p mode).

### 3.6. HPLC-DAD Quantification of Caffeoylquinic Acids

The HPLC analysis was performed on Schimadzu Nexera-I LC-2040C 3D Plus liquid chromatograph equipped with a photodiode array detector (Schimadzu, Tokyo, Japan) on analytical column Force C18 (150 × 4.6 mm, 3 µm) at a temperature of 30 °C. The elution was performed in a gradient mode using a mixture of 0.1% of formic acid in water (A) and methanol (B) as follows: 0 min, 20% B; 5 min, 20% B; 37 min, 60% B; 38 min, 80% B; 42 min, 80% B; 43 min, 20% B; 47 min, 20% B. The injection volume was 2 µL, the flow rate was 0.6 mL/min and runs were monitored at 320 nm. Before analysis, samples were filtered through 0.22 µm syringe filter. Chlorogenic acid with Rt 12.6 min (0.019–0.305 mg/mL, R^2^ 0.9999), 3,4-dicaffeoylquinic acid Rt 25.42 min (0.005–0.083 mg/mL, R^2^ 0.9999), 3,5-dicaffeoylquinic acid Rt 25.70 min (0.019–0.308 mg/mL, R^2^ 0.9999), 1,5-dicaffeoylquinic acid Rt 26.35 min (0.022–0.355 mg/mL, R^2^ 0.9999) and Rt 29.13 min (0.008–0.135 mg/mL, R^2^ 0.9999) were used as standards for preparation of the calibration curves (Figure 3). The experiment was performed in triplicate and the results are expressed as mg/g DE.

### 3.7. Evaluation of Total Phenolic (TPC) and Total Flavonoid (TFC) Contents

TPC was determined by Folin–Ciocalteu method [81] and the results were expressed as milligrams gallic acid equivalents per gram of dry extract (mg GAE/g DE). TFC was determined by Zhishen et al. colorimetric assay [82] and the results were expressed as milligrams of catechin equivalents per gram of dry extract (md CE/g DE).

### 3.8. Assessment of Antioxidant Potential

DPPH^●^ (1,1-diphenyl-2-picrylhydrazyl) and ABTS^●+^ scavenging activities were determined using the procedure described by Thaipong et al. [83] and the results were expressed as mM Trolox equivalents per a gram of dry extract. FRAP (ferric ion reducing antioxidant power was performed according to procedure described in [84] and the results were expressed as µM Fe^2+^/g DE.

### 3.9. SPF and UV-B Photoprotective Study

The assay was performed according to the procedure described by Bojilov et al. [80] using different concentrations (1000, 250, 125, 62.5 and 15.6 µg/mL) of the sample and standards (caffeic acid, chlorogenic acid, and rutin). The sunscreen percentage absorption based on SPF (UV-B) was calculated as described in [4]: UV-B% = 100 − (100/SPF).

### 3.10. Biofilm Inhibition and Assessment of Biofilm Viability

#### 3.10.1. Bacterial Strains and Growth Conditions

In this study, Gram-negative *Escherichia coli* 25922 and Gram-positive *Staphylococcus aureus* 29213 bacterial strains were utilized. The strains were obtained from the American Type Culture Collection (ATCC, Manassas, VA, USA), while *Pseudomonas aeruginosa* PAO1 was sourced from the International Reference Panel [85]. All bacterial strains were stored in 8% DMSO at −80 °C and maintained at 4 °C on Nutrient Agar for *E. coli* (HiMedia, Bedford, PA, USA) and Tryptic Soy Agar (TSA, Sigma, Burlington, MA, USA) for *S. aureus* and *Pseudomonas aeruginosa* slants. For screening procedures, the strains were cultivated in Nutrient Broth (HiMedia, USA) and Tryptic Soy Broth (TSA, Sigma, Burlington, MA, USA) at 37 °C for 18 h.

#### 3.10.2. Biofilm Inhibition

Overnight bacterial cultures were used as an initial inoculum to evaluate the anti-biofilm efficacy of *I. salicina* extract. The conditions and the analysis were the same as described in [23] using M63 media containing KH_2_PO_4_ (0.02 M), K_2_HPO_4_ (0.02 M), (NH_4_)_2_SO_4_ (0.02 M), MgSO_4_ (0.1 mM) and glucose (0.04 M). The bacterial inoculum diluted in M63 with 2% DMSO served as a control sample. The cultivation was performed for 24 h at 37 °C. Aqueous crystal violet (0.1%) was used for staining of the adherent bacteria and the optical density was measured at 570 nm. To confirm the quantitative data of biofilm inhibitions the experiment was performed in six replicates, and average values were calculated as percentages.

#### 3.10.3. Assessment of Biofilm Viability Using Live/Dead Staining

The biofilms were cultivated on sterilized borosilicate cover glasses for evaluation of the plant extract effects on bacterial viability. Further, the biofilm was stained with Live/Dead BacLight Bacterial Viability Kits (Invitrogen, Carlsbad, CA, USA) according to the producer’s instructions, mounted on microscopic slides using Fluoromount Mounting Medium (Sigma, USA) and observed on a Nikon Ti-U confocal laser scanning microscope in epifluorescence mode with 60× oil Plan Apo objective, at excitation wavelengths of 488 nm and 543 nm. NIS-Elements software and the Icy bio-imaging program were used for processing the acquired images.

#### 3.10.4. Scanning Electron Microscopy (SEM)

For SEM analysis, the biofilms were grown on sterile plastic pieces. The slides were then placed in 35 mm diameter Petri dishes, inoculated with bacterial suspension and addition of the extract, and allowed to develop biofilms over 24 h. After the incubation period, samples were washed with PBS and fixed for 4 h at 4 °C in 4% glutaraldehyde in a 0.1 M sodium cacodylate buffer (pH 7.2). Further, the samples were washed with cacodylate buffer and post-fixed with 1% OsO_4_ for 1 h, dehydrated via a graded ethanol series, coated with gold and observed on Lyra/Tescan scanning electron microscope (TESCAN GROUP a.s., Brno, Czech Republic), with at least 10 randomly selected digital images taken per sample, and two samples analyzed per variant.

## 4. Conclusions

In this study we attempted to obtain more comprehensive information on the chemical composition and biological properties of *Inula salicina.* Qualitative phytochemical analysis showed the presence of various phenolic compounds-hydroxycinnamic and hydroxybenzoic acids and their glycosides, acylquinic acids, caffeoylhexaric acids and flavonoids and their glycosides, among which chlorogenic and dicaffeoylquinic acids were the predominant compounds. In addition, a new natural compound, N-(8-methylnepetin)-3β-hydroxypiperidin-2-one was discovered. The presence of flavoalkaloids in the studied species is described for the first time in genus *Inula* and could be of chemotaxonomic significance.

The methanol extract of *I. salicina* exhibited good radical scavenging activity, high SPF and notable anti-biofilm effects, especially in *P. aeruginosa* attributed to the presence of phenolic compounds and especially of chlorogenic acid. Our findings indicated the potential application of *Inula salicina* extract as biofilm inhibitor, offering promising prospects for treating various surfaces contaminated with biofilms, such as implants or for directly treating superficial wounds. The extract may also be a good candidate for the development of cosmetic products. However, additional experiments on skin models and evaluation of the cytotoxicity of *I. salicina* extract are needed to assess its safety potential.

## Figures and Tables

**Table 1:** Identification of compounds by UHPLC-MS/MS in *Inula salicina* methanol extract.

No	Rt (min)	Compound Name	Formula	[M-H]^−^, *m*/*z*	Δ, ppm	MS/MS Fragments	Identification *
1	0.75	Quinic acid	C_7_H_11_O_6_	191.0554	1.64	191, **173**, 163, 145, 129, 115, 101	MS
2	3.27	Protocatechuic acid	C_7_H_5_O_4_	153.0183	−3.49	153, **109**	MS
3	4.14	Neochlorogenic acid (3-*O*-caffeoylquinic acid)	C_16_H_17_O_9_	353.0878	−0.10	353, **191**, 179, 135	MS, St
4	5.75	*O*-Caffeoyl hexose	C_15_H_17_O_9_	341.0879	0.16	341, **179**, 135	MS
5	6.07	3-*O*-*p*-Coumaroylquinic acid	C_16_H_17_O_8_	337.0929	−0.08	337, 191, **163**	MS
6	6.85	*O*-Caffeoyl hexose isomer	C_15_H_17_O_9_	341.0877	−0.29	341, 281, 251, 221, **179**, 161, 135	MS
7	6.97	Ferulic acid	C_10_H_9_O_4_	193.0499	−3.26	193, **178**, 149, 134	MS
8	7.47	Chlorogenic acid (5-*O*-caffeoylquinic acid)	C_16_H_17_O_9_	353.0881	0.76	353, **191**, 179, 135	MS, St, NMR
9	7.89	Caffeic acid	C_9_H_7_O_4_	179.0342	−1.08	179, **135**	MS, St
10	9.41	Chlorogenic acid isomer	C_16_H_17_O_9_	353.0877	−0.45	353, **191**, 179, 161	MS
11	9.95	Chlorogenic acid isomer	C_16_H_17_O_9_	353.0876	−0.62	353, **191**, 179, 161, 135	MS
12	10.15	4-*O*-*p*-Coumaroylquinic acid	C_16_H_17_O_8_	337.0934	1.55	337, 191, **173**, 163	MS
13	10.65	5-*O*-*p*-Coumaroylquinic acid	C_16_H_17_O_8_	337.0932	1.01	337, **191**, 173, 163	MS
14	11.76	*p*-Coumaric acid	C_9_H_7_O_3_	163.0388	−4.58	163, 135, **119**	MS
15	12.44	*p*-Coumaroylquinic acid isomer	C_16_H_17_O_8_	337.0933	1.28	337, **191**, 163	MS
16	12.55	5-*O*-Feruloylquinic acid	C_17_H_19_O_9_	367.1033	−0.58	367, **191**, 173	MS
17	17.81	Rutin (quercetin 3-*O*-rutinoside)	C_27_H_29_O_16_	609.1465	0.41	**609**, 301	MS, St, NMR
18	18.39	Isoquercitrin (quercetin 3-*O*-glucoside)	C_21_H_19_O_12_	463.0882	0.06	**463**, 301, 300,	MS, St, NMR
19	18.55	Luteolin 7-*O*-glucoside	C_21_H_19_O_11_	447.0933	0.09	**447**, 285, 284	MS, St, NMR
20	18.87	Patulitrin (patuletin 7-*O*-glucoside)	C_22_H_21_O_13_	493.0992	0.91	493, **331**, 330, 316	MS, NMR
21	19.35	Caffeoyl-(salicyl)-hexoside	C_22_H_21_O_11_	461.1089	−0.04	**461**, 323, 221, 179, 161, 137	MS
22	19.63	Nepitrin (nepetin 7-*O*-glucoside)	C_22_H_21_O_12_	477.1036	−0.51	477, 315	MS, NMR
23	19.66	3,4-Di-*O*-caffeoylquinic acid	C_25_H_23_O_12_	515.1191	−0.81	**515**, 353, 335, 191, 179, 173	MS, St
24	20.12	Isorhamnetin hexoside	C_22_H_21_O_12_	477.1038	−0.12	**477**, 315, 299	MS
25	20.56	3,5-Di-*O*-caffeoylquinic acid	C_25_H_23_O_12_	515.1191	−0.81	515, **353**, 191, 179	MS, St, NMR
26	20.67	1,5-Di-*O*-caffeoylquinic acid	C_25_H_23_O_12_	515.1191	−0.81	515, 353, **191**, 179	MS, St, NMR
27	20.87	Tricaffeoylhexaric acid	C_33_H_27_O_17_	695.1261	1.84	695, 533, 371, **209**, 191	MS
28	20.94	N-(8-methylnepetin)-3-hydroxypiperidin-2-one	C_22_H_20_O_9_N	442.1150	1.37	442, **327**, 312, 284, 256	MS, NMR
29	21.28	Kaempferol 3-*O*-glucoside (astragallin)	C_21_H_19_O_11_	447.0937	0.97	**447**, 285, 284, 255	MS, NMR
30	22.16	4,5-Di-*O*-caffeoylquinic acid	C_25_H_23_O_12_	515.1190	−1.05	515, **353**, 191, 179, 173	MS, St
31	22.19	3-*O-p*-Coumaroyl-4-*O*-caffeoylquinic acid	C_25_H_23_O_11_	499.1250	0.77	**499**, 353, 337, 335, 319, 173, 163	MS
32	22.20	Tricaffeoylhexaric acid isomer	C_33_H_27_O_17_	695.1261	1.84	695, 533, 371, **209**, 191	MS
33	22.36	3-*O*-Caffeoyl-4-*O-p*-coumaroylquinic acid	C_25_H_23_O_11_	499.1249	0.53	499, 353, 337, 335, 319, **173**, 163	MS
34	22.39	Tricaffeoylhexaric acid isomer	C_33_H_27_O_17_	695.1261	1.84	695, 533, 371, **209**, 191	MS
35	22.53	Tricaffeoylhexaric acid isomer	C_33_H_27_O_17_	695.1261	1.84	695, 533, 371, **209**, 191	MS
36	22.57	3-*O-p*-Coumaroyl-5-*O*-caffeoylquinic acid	C_25_H_23_O_11_	499.1252	1.26	499, 353, 337, 191, **163**	MS
37	22.67	3-*O*-Caffeoyl-5-*O-p*-coumaroylquinic acid	C_25_H_23_O_11_	499.1251	1.08	499, 353, 337, **191**, 179, 163	MS
38	22.79	Caffeoylferuloyl quinic acid	C_26_H_25_O_12_	529.1353	0.20	**529**, 367, 161	MS
39	23.03	Caffeoylferuloyl quinic acid isomer	C_26_H_25_O_12_	529.1357	1.01	529, 367, 353, **191**	MS
40	23.09	4-*O-p*-Coumaroyl-5-*O*-caffeoylquinic acid	C_25_H_23_O_11_	499.1250	0.89	499, 337, 191, **173**, 163	MS
41	23.20	4-*O*-Caffeoyl-5-*O-p*-coumaroylquinic acid	C_25_H_23_O_11_	499.1250	0.83	499, **353**, 337, 191, 179, 173	MS
42	23.34	Caffeoylferuloyl quinic acid isomer	C_26_H_25_O_12_	529.1352	0.08	**529**, 367, 179, 161	MS
43	23.52	3,5-di-*O-p*-Coumaroylquinic acid	C_25_H_23_O_10_	483.1298	0.19	483, **337**, 319, 191, 163	MS
44	23.66	Tetracaffeoylhexaric acid	C_42_H_33_O_20_	857.1575	1.11	857, 695, 533,371, 209, 191	MS
45	23.69	Caffeoylferuloyl quinic acid isomer	C_26_H_25_O_12_	529.1353	0.20	**529**, 367, 179, 161	MS
46	23.76	2-Methylbutanoyl/isovaleryl dicaffeoylhexaric acid	C_29_H_29_O_15_	617.1505	−1.1	617, 455, **293**, 191, 179	MS
47	23.81	Quercetin	C_15_H_9_O_7_	301.0353	−0.22	**301**, 179, 151	MS, St
48	23.85	Luteolin	C_15_H_9_O_6_	285.0404	−0.20	**285**	MS, St, NMR
49	23.89	4,5-di-*O*-*p*-Coumaroylquinic acid	C_25_H_23_O_10_	483.1298	0.19	483, **337**, 191, 173, 163	MS
50	23.91	3,4,5-Tricaffeoylquinic acid	C_34_H_29_O_15_	677.1516	1.34	677, **515**, 353, 335, 191, 179, 173, 161	MS
51	23.95	Patuletin (6-methoxyquercetin)	C_16_H_11_O_8_	331.0459	−0.22	**331**, 316, 287, 271	MS, NMR
52	24.18	Nepetin (6-methoxyluteolin)	C_16_H_11_O_7_	315.0511	0.29	315, 301, **300**	MS, NMR
53	24.32	Isobutanoyl tricaffeoylhexaric acid	C_37_H_33_O_18_	765.1679	1.59	765, 603, 441, **279**, 261, 191	MS
54	24.85	2-Methylbutanoyl/isovaleryl tricaffeoylhexaric acid isomer	C_38_H_35_O_18_	779.1830	0.80	779, 617, 455, **293**, 275, 191	MS
55	25.12	Chrysoeriol	C_16_H_11_O_6_	299.0563	0.75	299, **284**	MS
56	25.31	Jaceosidin	C_17_H_13_O_7_	329.0667	−0.08	329, **314**, 299	MS
57	25.49	Quercetagetin trimethyl ether	C_18_H_15_O_8_	359.0773	0.25	359, **344**, 329, 301	MS
58	25.99	Apigenin	C_15_H_9_O_5_	269.0457	0.63	**269**	MS, St, NMR

* MS—The compounds were tentatively identified; St—the identity of the compounds was confirmed by injecting authentic samples; NMR—the compounds were isolated and their identity was confirmed by NMR experiments.

**Table 2:** ^1^H (600 MHz) and ^13^C (150 MHz) NMR spectroscopic data of N-(8-methylnepetin)-3-hydroxypiperidin-2-one (**28**) recorded in CD_3_OD (δ in ppm, multiplicity, *J* in Hz).

№	δ_H_	δ_C_ *	№	δ_H_	δ_C_ *
2		164.5	3′		145.8
3	6.59 (s)	101.9	4′		150.3
4		182.5	5′	6.94 (d, 8.4)	115.6
5		168.5	6′	7.45 (dd, 2.2, 8.4)	119.0
6		132.5	OMe	3.89 (s)	59.6
7		160.5	2”		172.4
8		97.3	3”	4.07 (dd, 5.5, 9.3)	69.3
9		152.5	4″	2.18 (m)/2.48 (m)	28.8
10		103.1	5″	2.09 (m)/1.95 (m)	23.0
1′		122.1	6″	3.57 (m)/3.36 (m)	53.4
2′	7.62 (d, 2.2)	113.5	7″	4.58 (d, 13.3)/4.72 (d, 13.3)	47.4

* Deduced from HSQC and HMBC experiments.

**Table 3:** Content of total phenolics (TPC), total flavonoids (TFC) and individual compounds, and antioxidant potential (DPPH, ABTS and FRAP) of *I. salicina*.

	TPC ^a^	TFC ^b^	5-CQA ^c^	3,4-DCQA ^c^	3,5-DCQA ^c^	1,5-DCQA ^c^	4,5-DCQA ^c^	DPPH ^d^	ABTS ^d^	FRAP ^e^
Mean	215.57	87.44	103.39	5.38±	30.62	18.58	23.33	0.741	0.711	5.77
SD	2.52	0.52	1.30	0.26	0.79	0.69	0.50	0.006	0.007	0.08

^a^ expressed as mg GAE/g DE; ^b^ expressed as mg CE/g DE; ^c^ expressed as mg/g DE; ^d^ expressed as mg TE/g DE; ^e^ expressed as mg/g DE µM Fe^2+^/g DE; Mean and SD from 3 measurements.

**Table 4:** The gradient of the chromatographic separation.

Time (min)	Solvent A * (%)	Solvent B * (%)
0→1	95	5
1→20	95→82	5→18
20→24	82→60	18→40
24→27	60→30	40→70
27→29	30→5	70→95
29→31	5	95

* A: 0.1% formic acid in water; B: 0.1% formic acid in acetonitrile.

## Data Availability

Data are contained within this article.

## Supplementary Materials

The following supporting information can be downloaded at https://www.mdpi.com/article/10.3390/ph17070844/s1, Table S1: ^1^H NMR data of caffeoylquinic acids; Table S2: ^1^H NMR data of flavonoid aglycones; Table S3: ^1^H NMR data of flavonoid glycosides; Figure S1: Structures of the isolated compounds; Figure S2: Full scan mass spectrum of compound **28** in positive ionization mode; Figure S3: ^1^H NMR spectrum of **28**; Figure S4: COSY spectrum of **28**; Figure S5: HSQC spectrum of **28**; Figure S6: HMBC spectrum of **28**; Figure S7: UV spectrum of **28**.
