# Supplementary material for: Inula salicina L.: Insights into Its Polyphenolic Constituents and Biological Activity
Source: Pharmaceuticals (Basel). 2024 Jun 27;17(7):844. doi: 10.3390/ph17070844 (PMC11279402; doi:10.3390/ph17070844)
Supplement: Supplementary file 1 [file pharmaceuticals-17-00844-s001.zip › pharmaceuticals-3061729-supplementary.pdf]

SUPPLEMANTARY MATERIAL

Inula salicina L.: Insights into Its Polyphenolic Constituents and Biological Activity

Viktoria Ivanova[1], Paraskev Nedialkov[2], Petya Dimitrova[3], Tsvetelina Paunova-Krasteva[3], Antoaneta Trendafilova[1*]

[1] *Institute of Organic Chemistry with Centre of Phytochemistry, Bulgarian Academy of Sciences, 1113 Sofia, Bulgaria*
[2] *Pharmacognosy Department, Faculty of Pharmacy, Medical University of Sofia, 1000 Sofia, Bulgaria*
[3] *Stephan Angeloff Institute of Microbiology, Bulgarian Academy of Sciences, 1113 Sofia, Bulgaria*
*Corresponding author (Antoaneta.Trendafilova@orgchm.bas.bg)

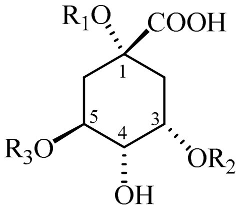

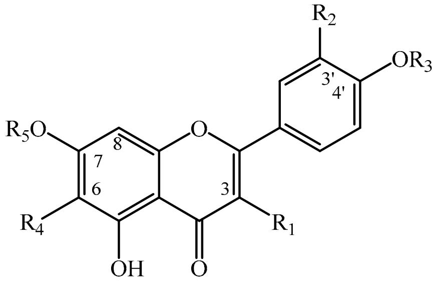

	R_1	R_2	R_3
**5-CQA (8)**	H	H	Caff
**3,5-DCQA (25)**	Caff	H	Caff
**1,5-DCQA (26)**	H	Caff	Caff

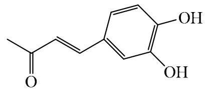

Caff

17 R_1=OGlc-Rha R_2=OH, R_3=R_4=R_5=H
18 R_1=OGlc, R_2=OH, R_3=R_4=R_5=H
19 R_1=R_3=R_4=H, R_2=OH, , R_5=Glc
20 R_1=R_2=OH, R_3=H, R_4=OCH_3, R_5=Glc
22 R_1=R_3=H, R_2=OH, R_4=OCH_3, R_5=Glc
29 R_1=OGlc, R_2=R_3=R_4=R_5=H
47 R_1=R_2=OH, R_3=R_4=R_5=H
48 R_1=R_3=R_4=R_5=H, R_2=OH
51 R_1=R_2=OH, R_3=H, R_4=R_5=OCH_3
52 R_1=R_3=R_5=H, R_2=OH, R_4=OCH_3
58 R_1=R_2=R_3=R_4=R_5=H

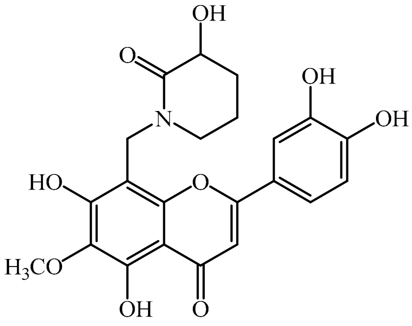

28

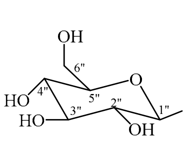

Glc

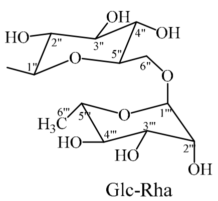

Glc-Rha

Figure S1. Structures of the isolated compounds

Table S1. ^{1}H NMR data of caffeoylquinic acids (600 MHz, CD_3OD, δ (ppm), *J* (Hz))

H	**Chlorogenic acid (8)**	**3,5-dicafeoyquinic acid (25)***	**1,5-dicaffeoylquinic acid (26)***
2	2.16 (dd, 5.2, 14.4)<br>2.04 (dd, 3.4, 14.4)	2.00 (dd, 3.0, 15.0)<br>2.16 (dd, 3.2, 15.0)	1.98 (dd, 3.5, 14.5)<br>2.12 (dd, 3.2, 14.5)
3	4.16 (ddd, 3.2, 3.4, 5.2)	5.40 (ddd, 3.0, 3.2, 3.8)	4.21 (td, 3.0, 3.2, 3.8)
4	3.72 (dd, 3.2, 8.6)	3.96 (dd, 3.8, 9.5)	3.71(dd, 3.8, 9.5)
5	5.30 (ddd, 3.2, 8.6, 9.7)	5.37 (ddd, 3.8, 9.5, 10.8)	5.43 (ddd, 3.5, 9.5, 10.8)
6	2.06 (dd, 9.7, 13.3)<br>2.23 (dd, 3.2, 13.3)	2.19 (dd, 3.8, 13.2)<br>2.23 (dd, 10.8, 13.2)	2.26 (dd, 3.5, 13.4)<br>2.65 (dd, 10.8, 13.4)
2'	7.05 (d, 2.1)	7.06 (d, 1.5)	7.05 (d, 2.0)
5'	6.77 (d, 8.2)	6.78 (d, 8.1)	6.76 (d, 8.1)
6'	6.93 (dd, 2.1, 8.2)	7.00 (dd, 1.5, 8.1)	6.94 (dd, 2.0, 8.0)
7'	7.55 (d, 15.9)	7.48 (d, 16.0)	7.56 (d, 16.0)
8'	6.25 (d, 15.9)	6.25 (d, 16.0)	6.31 (d, 16.0)

*- The signals for the second caffeoyl residue are identical to the first one

Table S2. ^{1}H NMR data of flavonoid aglycones (600 MHz, CD_3OD, δ (ppm), *J* (Hz))

H	**Quercetin (47)**	**Luteolin (48)**	**Patuletin (51)**	**Nepetin (52)**	**Apigenin (58)**
3		6.53 (s)		6.53 (s)	6.58 (s)
6	6.18 (d, 2.1)	6.18 (d, 1.5)		-	6.20 (d, 2.0)
8	6.35 (d, 2.1)	6.42 (d, 1.5)	6.51 (s)	6.52 (s)	6.45 d, 2.0)
2'	7.67 (d, 2.2)	7.37 (d, 2.0)	7.72 (d, 2.2)	7.36 (d, 2.0)	7.80 (d, 8.2)
3'	-	-		-	6.90 (d, 8.2)
5'	6.85 (d,8.4)	6.89 (d, 8.2)	6.86 (d, 8.5)	6.88 (d, 8.2)	6.90 (d, 8.2)
6'	7.58 (dd, 2.2, 8.4)	7.38 (dd, 2.0, 8.2)	7.60 (dd, 2.2, 8.5)	7.38 (dd, 2.0, 8.2)	7.80 (d, 8.2)
OMe		-	3.87 (s)	3.86 (s)	

Table S3. ^{1}H NMR data of flavonoid glycosides (600 MHz, CD_3OD, δ (ppm), *J* (Hz))

H	**Rutin (17)**	**Isoquercitrin (18)**	**Luteolin-7-*O*-glucoside (19)**	**Patulitrin (20)**	**Nepitrin (22)**	**Astragalin (29)**
3	-	-	6.58 (s)		6.58 (s)	-
6	6.24 (d, 2.0)	6.19 (d, 2.0)	6.48 (d, 2.1)		-	6.23 (d, 2.1)
8	6.44 (d, 2.0)	6.39 (d, 2.0)	6.78 (d, 2.1)	6.91 (s)	6.92 (s)	6.43 (d, 2.1)
2'	6.90 (d, 2.0)	7.57 (d, 2.1)	7.38 (d, 2.1)	7.70 (d, 2.2)	7.38 (d, 2.2)	8.08 (d, 8.9)
3'	-	-			-	6.91 (d, 8.9)
5'	6.69 (d, 8.5)	6.83 (d,8.5)	6.84 (d,8.3)	6.88 (d, 8.5)	6.88 (d, 8.0)	6.91 (d, 8.9)
6'	7.64 dd, (2.0, 8.5)	7.58 (dd, 2.1, 8.5)	7.42 (dd, 2.1, 8.3)	7.52 (dd, 2.2, 8.5)	7.40 (dd, 2.2, 8.0)	8.08 (d, 8.9)
OMe				3.80 (s)	3.88 (s)	-
1''	5.10 (d, 7.5)	5.24 (d ,7.6)	5.08 (d, 7.5)	5.10 (d, 7.2)	5.11 (d, 7.5)	5.28 (d, 7.5)
2''	3.45 dd (7.5, 9.1)	3.47 (dd, 7.6, 9.0)	3.48 (dd, 7.5, 9.0)	3.42 (dd, 7.2, 9.0)	3.57 (dd, 7.5, 9.3)	3.43 (dd, 7.5, 9.1)
3''	3.45 m	3.42 (m)	3.41 (m)	3.30 (m)	3.52 (m)	3.56 (m)
4''	3.33 m	3.34 (t, 9.0)	3.34 (t, 9.0)	3.38 (t, 9.0)	3.41 (t, 9.3)	3.43 (t, 9.1)
5''	3.44m	3.22 (m)	3.51 (m)	3.51 (m)	3.58 (m)	3.23 (m)
6a''	3.80 dd (2.5, 11.8)	3.70 dd (2.3, 12.1)	3.90 dd (2.3, 12.3)	3.88 dd (2.2, 12.3)	3.94 (dd, 2.2, 12.2)	3.72 (dd, 2.2, 12.0)
6b''	3.53 dd (6.7, 11.8)	3.56 dd (5.6, 12.1)	3.71 dd (5.6, 12.3)	3.68 dd (5.6, 12.3)	3.72 (dd, 6.1, 12.2)	3.54 (dd, 5.5, 12.0)
1'''	4.51 (d, 1.8)					-
2'''-5'''	3.40-3.90 (4H, m)					
6'''	1.11 d (6.5)					

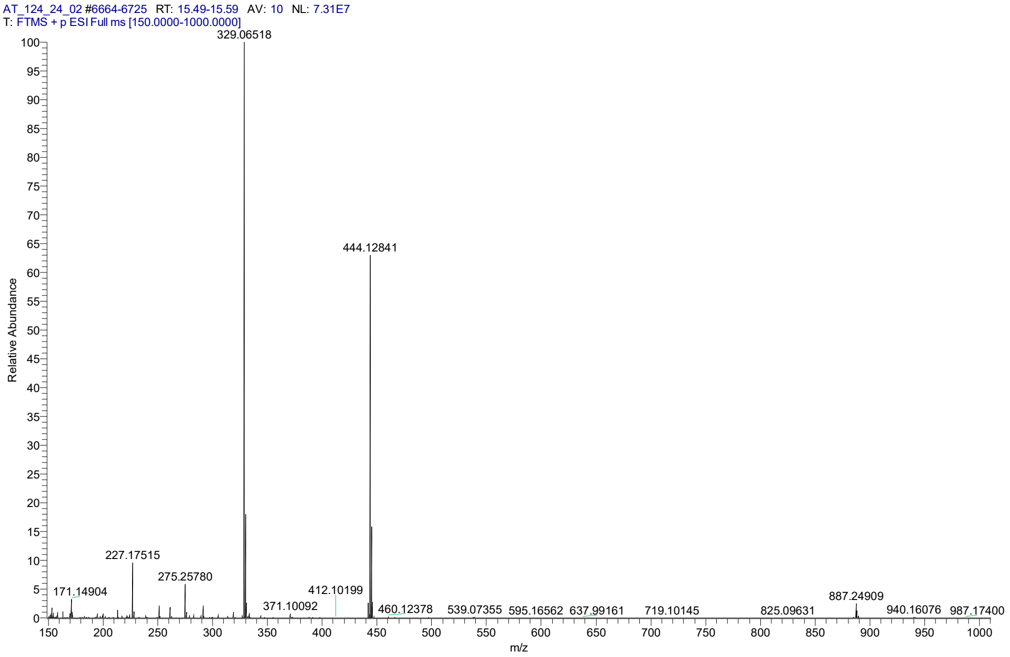

Figure S2. Full scan mass spectrum of compound **28** in positive ionization mode.

m/z	Theo. Mass	Delta (ppm)	RDB equiv.	Composition
444.12841	444.12891	-1.12	12.5	C22 H22 O9 N

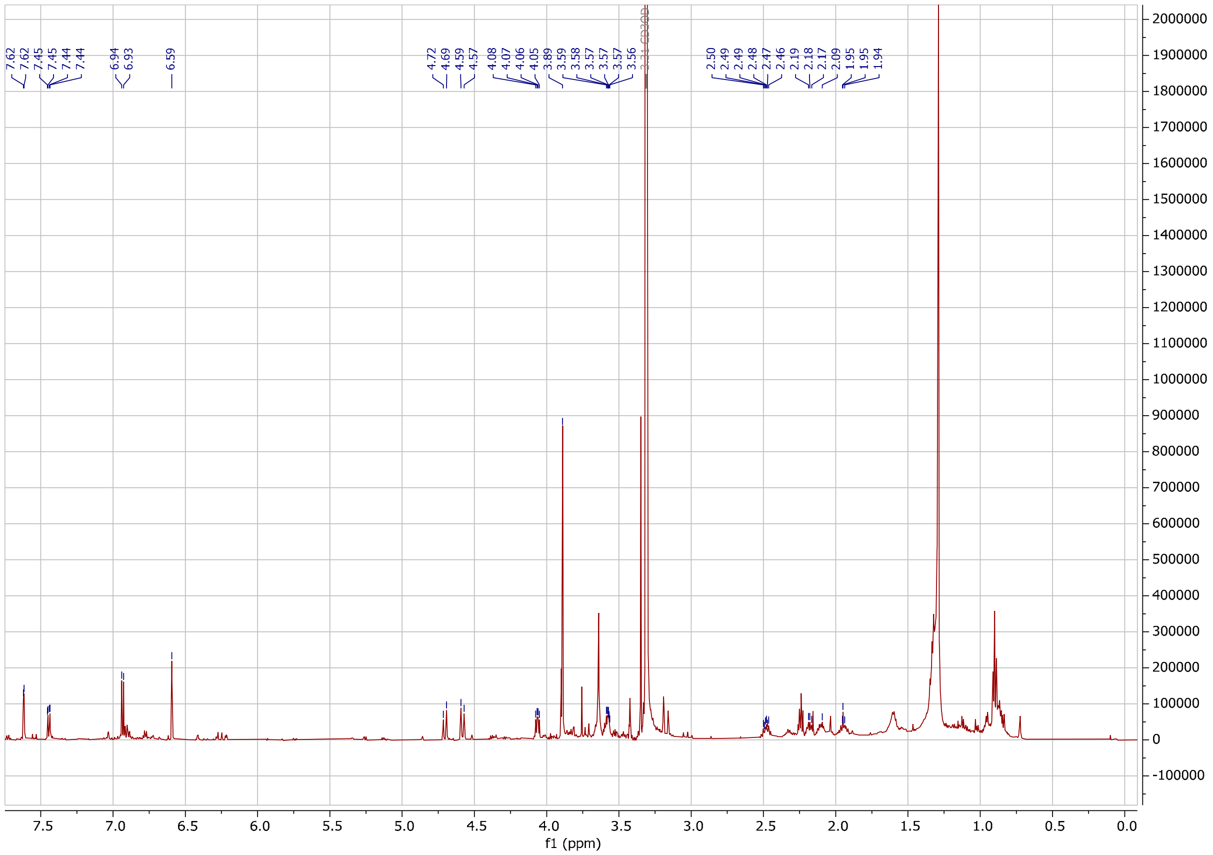

Figure S3: ^{1}H NMR spectrum of compound **28**

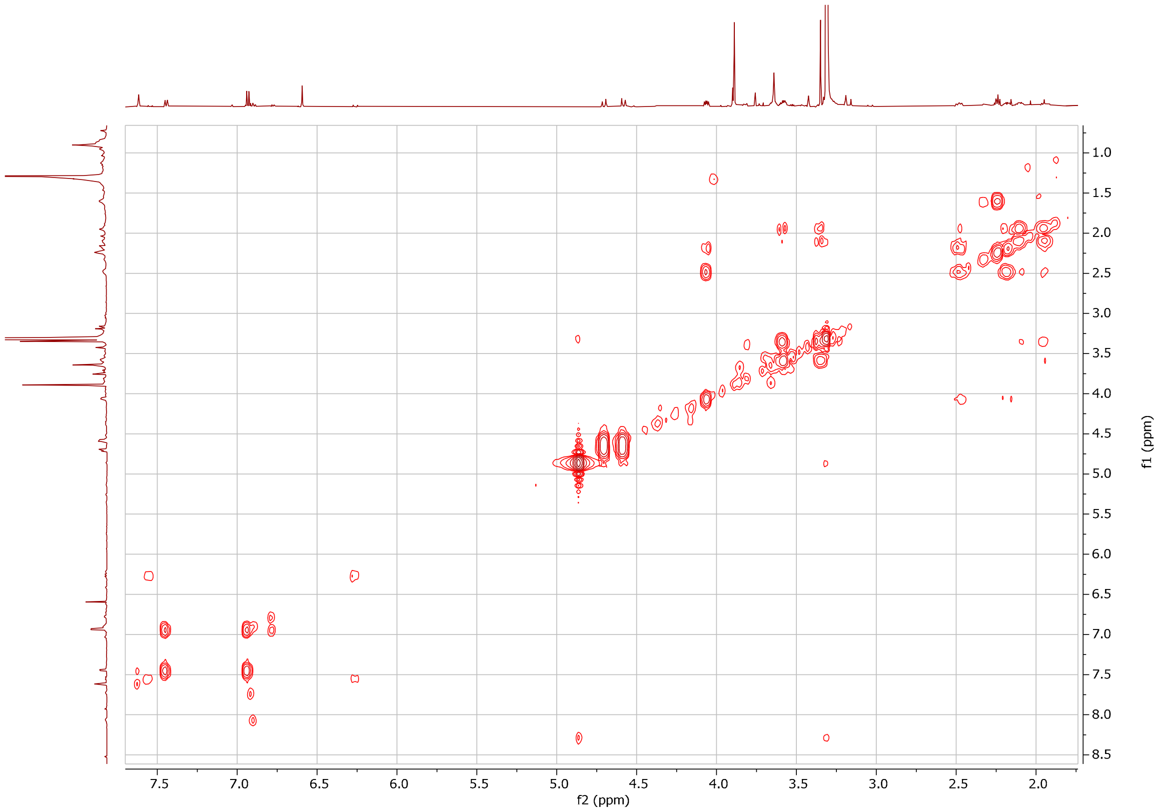

Figure S4: COSY spectrum of compound **28**

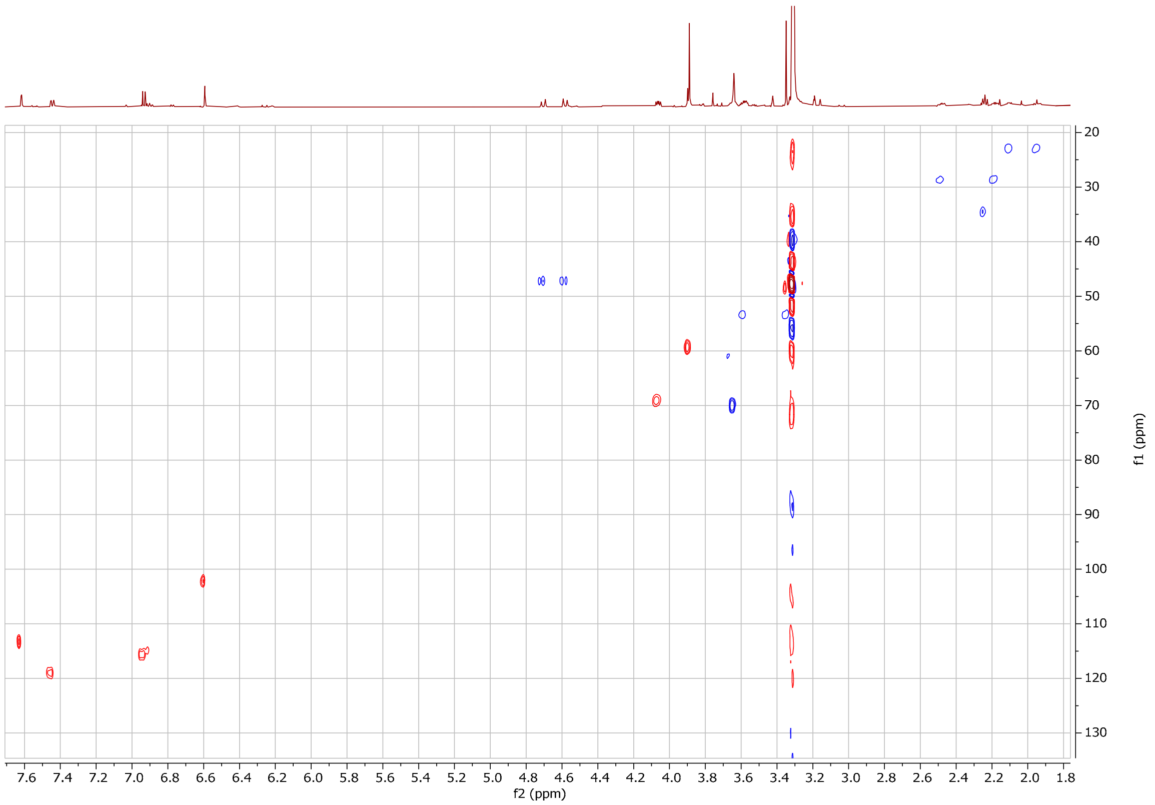

Figure S5: HSQC spectrum of compound **28**

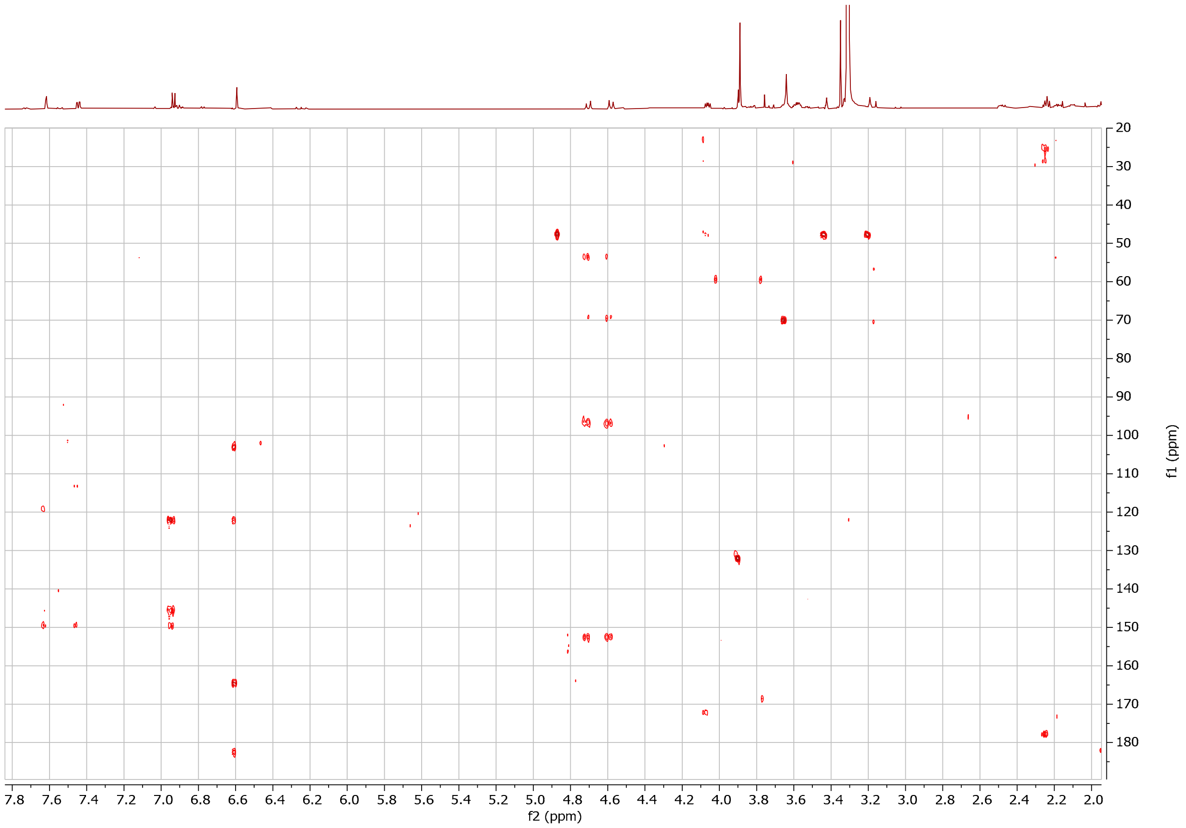

Figure S6: HMBC spectrum of compound **28**

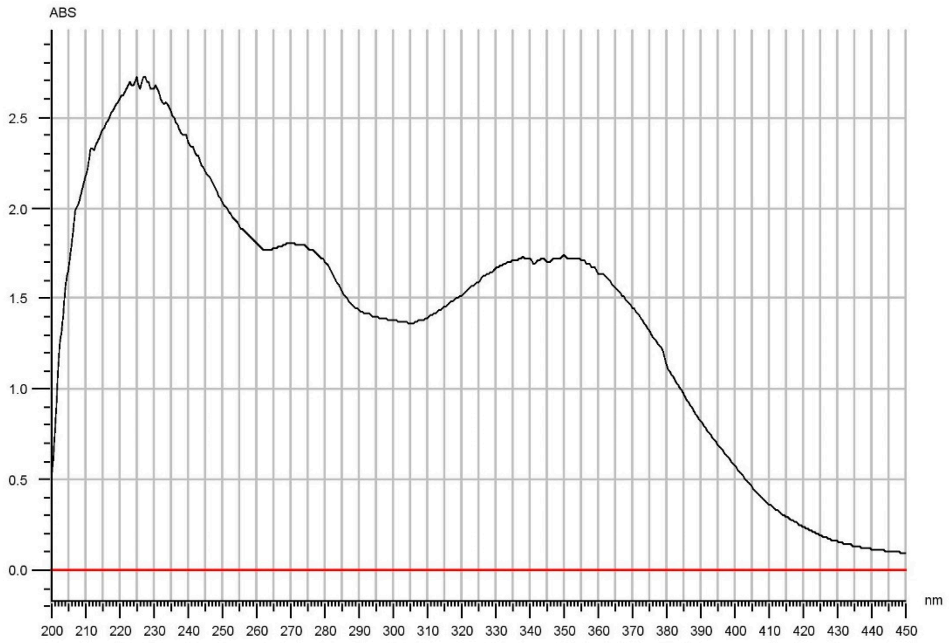

Figure S7: UV spectrum of compound **28**
